# The Burden of Cost in Bronchiolitis Obliterans Syndrome: Predictions for the Next Decade

**DOI:** 10.36469/9815

**Published:** 2016-06-09

**Authors:** Christopher A. Jones, David G. Chapman, Peter Weimersheimer, Luca Fernandez, Oscar Alejandro Mesa, Christian Peters, Bart M. Vanaudenaerde, Mitchell C. Norotsky, Robin Vos

**Affiliations:** 1 Department of Surgery Vermont Cancer Center; Global Health Economics Unit of the Vermont Center for Clinical and Translational Science; University of Vermont College of Medicine, Burlington, Vermont, USA; 2 Woolcock Institute of Medical Research, Sydney Medical School Vermont Lung Center; University of Sydney, New South Wales, Australia; 3 Department of Surgery University of Vermont College of Medicine, Burlington, Vermont, USA; 4 Global Health Economics Unit of the Vermont Center for Clinical and Translational Science; 5 Therakos, Inc., West Chester, PA, USA; 6 KU Leuven - University of Leuven, Department of Clinical and Experimental Medicine, Laboratory for Respiratory Diseases, Lung Transplant Unit; University Hospitals Leuven, Department of Respiratory Diseases, Lung Transplant and Respiratory Intermediate Care Unit, Leuven, Belgium

**Keywords:** bronchiolitis obliterans syndrome

## Abstract

In health economics, costs can be divided into both direct and indirect categories. Direct costs tend to consist of medical costs, which are those directly attributed to health care interventions (e.g., hospitalizations, pharmaceuticals, devices), and non-medical direct costs such as monitoring and professional caregiving. Indirect costs tend to comprise those related to lost productivity due to illness (or treatment), burden on systems outside of the healthcare domain, and other costs that can sometimes outweigh the entire sum of direct healthcare costs.

The most common life-threatening complication of lung and hematopoietic stem-cell transplantation (HSCT) is bronchiolitis obliterans syndrome (BOS). BOS is currently diagnosed as a 20% decline in the forced expiratory volume in one second (FEV1) from the best (baseline) post-transplantation value, and is a major cause of morbidity and mortality amongst lung and stem cell transplant patients. BOS affects half of all lung transplant patients within the first 5 years post-transplant, rising to the majority of patients (~80%) within the first decade following transplant.

We estimated both direct and indirect costs for the first 10 years following BOS diagnosis, a viewpoint that highlights a tremendous imbalance between healthcare and non-healthcare costs. The lost workforce resulting from BOS-related infirmity will cost society more than $3.7 Billion over the next decade, a figure that is more than double the estimated 10-year cost of treating BOS ($1.4B), including diagnostics, immunosuppressives, and additional complications. As such, BOS is estimated to present a burden of cost that must be evaluated in a new light to include the wider societal perspective.

## INTRODUCTION

Of all the present-day solid organs and tissues that can be transplanted, lungs and blood-producing stem cells present much higher rates of complication and rejection, both immediately following surgery and throughout the patient's life. The most common life-threatening complication of lung and hematopoietic stem-cell transplantation (HSCT) is a disease called bronchiolitis obliterans syndrome (BOS) in which the bronchioles proximal to the terminal airways become obstructed or obliterated due to airway narrowing.[1] BOS is currently diagnosed as a 20% decline in the forced expiratory volume in one second (FEV1) from the best (baseline) post-transplantation value, and is a major cause of mortality amongst lung and stem cell transplant patients.[2] Severity of the disease is further characterized by successive stages based on the extent of lung function abnormality, where FEV1 measurements of FEV1 are 81-90% (potential BOS), 66-80% of baseline (Stage 1), 51-65% of baseline (Stage 2) or ≤ 50% of baseline (Stage 3). BOS affects 50% of all lung transplant patients within the first 5 years post-transplant and this rises to 80% within 10 years of transplant.[3,4]

These proportions illustrate the progressive nature of BOS but this condition also presents at least three challenges to elucidating its total costs and associated impact on the health care budget. Firstly, whereas rates of acute rejection have declined with the practice of immunosuppression interventions, the rates of chronic rejection leading to BOS have not similarly improved.[5] Secondly, the lungs can develop BOS secondary to transplantation of other organ systems. As such, BOS becomes a major risk arising from chronic rejection of HSCT, where it is known to occur with a prevalence of 5.5%.[3,4,6] Thirdly, while proportionally fewer HSCT patients will develop BOS as compared with lung transplant patients, the use of HSCT is increasing, as is the annual rate of lung transplantations being performed word-wide, with a concomitant increase in the absolute numbers of transplant-related BOS. Thus, estimating the cost of BOS requires a thorough look at multifactorial, time-delayed events in a heterogeneous population with overlapping clinical pathways.[7,4,8]

Given this complexity, it comes as little surprise that there have been few published studies focusing on the economic burden of BOS. Several studies have evaluated the cost-effectiveness of lung transplantation more broadly, with world-wide estimates ranging from $30 000 to $150 000 USD per quality-adjusted life year (QALY), ranges that vacillate between acceptable and unacceptable cost-effectiveness thresholds, depending on the setting.[9,10,11,12,13] Most of these cost-effectiveness studies used healthcare costs as the numerator and QALYs as the denominator, yielding what is termed a cost-utility ratio.[15,16] While this approach is good for planning allocation of scarce healthcare resources, it fails to capture the time burden that is required to care for transplant recipients and BOS patients, in particular, as well as lost work time or lost opportunities for paid employment.[17] For example, a study comprising 53 lung transplant patients followed for 18 months in Holland reported that follow up healthcare was $600 USD/week (73%) higher in patients with BOS as compared to those without BOS.[14] This elevated cost was predominantly associated with length of hospitalization and increased medication use. The development of BOS has also been reported to require approximately an additional 2-7 hospital days.[14] From a health care services perspective, therefore, average BOS estimates can be multiplied by the average hospital and pharmaceutical costs that are incurred, using charge and billing records. However, BOS also affects social, labor, education, and other human capital values that benefit society.[18,19,20,21] As we illustrate in this study, the majority of the BOS burden arises in the counterfactual, i.e. what would have been possible without the disease in terms of opportunities for paid employment over the remainder of a patient's disease-free life.

In the case of HSCT, a cost-benefit analysis of hematopoietic stem cell patients in the United Kingdom reported that hospital readmission costs in those with graft-versus-host-disease (GvHD), a risk factor for BOS, were double as compared to those without GvHD.[21] Such equated to an additional $25 000 (£15 000) in costs. While these studies have not defined the burden outright, they set an excellent groundwork for estimating the complex economic burden of BOS.

## METHODS

### Data Sources

Within a broad interpretation of the value of a statistical life, we estimated a BOS-specific societal burden of cost resulting from a conservative combination of lost human capital and added medical expenditures.[23] From this wider societal perspective, total cost surrounding BOS over the next decade was evaluated based on the formula: Total Cost (TC) = Prevalent Population (PP) * (Medical Cost (MC) + Lost Wages (LWT)) * time (t in years).

The data populating this formula were derived from several population-based databases and secondary sources, as thusly described. Transplant statistics were gathered using data from both the United Network for Organ Sharing at the Health Resources and Services Administration (HRSA) and the Leukemia and Lymphoma Society (LLS). Prevalence of BOS, time delay to onset treatment costs, family and caregiver costs and average wage assumptions for the United States (SSA.gov) were summated and projected over a ten-year time horizon for the predicted BOS population in 2025.[3,4,24,25,26,27] Inflationary adjustments were applied based on indices reported by the Bureau of Labor Statistics (BLS).[28]

In our estimate of the wider societal burden of the disease, BOS patients were conservatively (at the outer bound of the model) assumed to have received a diagnosis within 5 years post-transplant, with approximately 25-30% being employed prior to transplant.[29,30] This is not to overlook the possibility that unemployment prior to BOS may have also been due to a loss of functional ability. We used the latter figure of 30% prior employment for our 10-year prospective calculations, but incorporated a range down to 25% in our sensitivity analysis.

Whereas physical functioning may restore to normative ranges following successful transplant, few BOS patients ultimately are able to return to the work force.[31] However, while the literature shows that BOS patients are unlikely to return to normative levels of physical and psychological functioning there is little to suggest that this is due to any increase in disability attributed to BOS.[32,33] In fact, one study has shown that even psychological stress impacting employability is not significantly increased by BOS over initial lung transplantation.[34] Instead, the impact of BOS on employment is caused by BOS representing a major cause of mortality amongst lung transplant recipients. Therefore, our sensitivity analysis included estimates of work years lost based on a range of employment outcomes, while our baseline calculation used the average for lung transplantation.

### Analysis

The LLS report titled, "Blood Marrow and Stem Cell Transplantation" provides an incidence of 7000 HSCTs in North America during 2013.[25] With a 5-year 5.5% incidence rate of BOS amongst HSCTs, this creates 385 incident patients every year.[26] The incidence of lung transplants was adapted from statistics reported by UNOS, and the International Society for Heart & Lung Transplantation (ISHLT). A 5-year 50% occurrence of BOS was applied to populations at risk for BOS, yielding an annual average of 904.4 diagnoses that are consequent to lung transplants. This figure is based on a total of 4522 cases of BOS observed over five-years, specifically from lung transplants.[26] Added to this is the average of 385 incident cases of BOS resulting from HSCT. In both cases, HSCT and lung transplant, five years of incidence data were used to derive a 2015 combined prevalence that accounted for mortality. For the purposes of creating a ten year burden of cost, this prevalence was then added to 10 years of incidence statistics for both sources of BOS patients.

### Sensitivity Analysis

A sensitivity analysis was performed to test the robustness of our assumptions. There is no clear consensus on how long a BOS survivor may take before returning to work, other than the average time to mortality (Dudek et al., 2003), nor is the average age of BOS patients clear (perhaps this is documented in ISHLT registry data slides/website, cf. my previous email), aside from using the average age of lung transplant recipients (HRSA).[36,24] Therefore, we varied the average duration of sickness for survivors, the average years lost to mortality, the average employment rate prior to lung transplant and the percentage of survivors who are able to ultimately return to the work force. As table two shows, only the average number of years lost to mortality—which reflects the average age of patients—has a strong influence on the outcome. This is because the number of survivors is so low that their ability to return to the workforce has a negligible impact on the vast overall costs of BOS. A lesser but still influential relationship is show when using the low estimate in the literature for prior employment.

## RESULTS

A total of 14 771 BOS patients were estimated from the last 5 years of trends reported in the registries to require treatment over the next decade (from 2015 to 2025). These patients will accrue a total of 82 744 years of lost wages. This lost workforce will cost society $3714 235 976.76 ($3.7B) over the next decade, a figure that is more than 2.7X ($3.7B vs. $1.3B) the estimated ten-year cost of treating BOS ($1353 120 580), including diagnostics, immunosuppressives, and additional complications.[14] As such, BOS is estimated to present a conservative burden of cost that will exceed $5 billion over the next decade in the United States alone (See Table 1). The sensitivity analysis shows that the average age of the patient, which is reflected in the number of work years lost to mortality, has the highest effect on outcomes. A 10-year change in the average years lost results in a 40% change in total burden of cost—without changing the medical cost.

**Table 1.** Present and 10-year Burden of Cost for BOS

| **Calculation** | | | |
|---|---|---|---|
| | **HCT** | **Lung** | **Total** |
| Estimated Prevalence | 608 | 1433 | 2041 |
| Estimated Incidence | 385 | 847 | 1232 |
| Ten Year Incidence | 3850 | 8880 | 12 730 |
| Total | 4458 | 10 313 | 14 771 |
| Statistical Years of Lost Wages | 82 744 | | |
| Ten Year Medical Cost | $1353 120 580.63 | | |
| Lost Wages | $3714 235 976 | | |
| Total[1] | $5067 356 557 | | |

[1]Only 30% of *total* lung transplant patients are reported as being employed prior to lung transplant (Singer et al., 2014).

**Table 2.** Sensitivity Analysis

| Analysis | | Years Lost | Wages Lost | Total W/ Medical Cost | % Total Change |
|---|---|---|---|---|---|
| **Baseline** | | | | | |
| | | 82 744 | $3714 235 976 | $5067 356 557 | n/a |
| **Prior Employment** | | | | | |
| | 0.25 | 54 859.30 | $2462 533 400.60 | $3815 653 981.23 | 24.701% |
| **Percent Disabled Survivors** | | | | | |
| | 0.75 | 81 716.35 | $3668 096 999.65 | $5021 217 580.28 | -0.911% |
| | 0.50 | 76 577.03 | $3437 402 114.12 | $4790 522 694.75 | -5.463% |
| | 0.25 | 71 437.70 | $3206 707 228.58 | $4559 827 809.21 | -10.016% |
| **Years Lost to Mortality** | | | | | |
| | 10 | 42 445.27 | $1905 290 483.97 | $3258 411 064.60 | -35.698% |
| | 30 | 126 401.41 | $2744 180 976.83 | $4097 301 557.46 | 38.637% |
| | 50 | 172 980.63 | $7764 782 384.87 | $9117 902 965.50 | 79.934% |
| **Survivors' Duration of Sickness** | | | | | |
| | 4 | 83 211.44 | $3735 208 239.08 | $5088 328 819.71 | 0.414% |
| | 5 | 83 445.04 | $3745 694 370.24 | $5098 814 950.87 | 0.621% |
| | 6 | 83 678.65 | $3756 180 501.40 | $5109 301 082.03 | 0.828% |

## DISCUSSION

Lung transplantation has become a viable treatment option for many end-stage lung diseases such as cystic fibrosis, chronic obstructive pulmonary disease (COPD), idiopathic pulmonary fibrosis and alpha-1 antitrypsin deficiency. While solid organ transplants have generally evolved in terms of donor-match efficiency and infection prevention, donor lungs still presents much higher rates of complication and rejection than other solid organ transplants, both immediately following surgery and throughout the patient's life. Moreover, the delayed-onset nature of BOS means that its prevalence overshadows its incidence, which leads to a surprising number of cases that exceed what would otherwise be expected from HSCT and lung transplant statistics in a mere one-year snapshot.

We compared contributors to the total cost of BOS as well as the sensitivity surrounding our assumptions. We counted lung transplants as having a uniform cost for a single lung; inclusion of double lung transplants would intensify our conclusions. As expected, the average age of patients had a strong impact on our results. Even extreme variation in patient recovery time and ability to return to work has little impact on the overall cost of BOS, because of the severe mortality associated. We found that even conservative estimates of collective lost work time can trump the medical cost of treating BOS in the decade ahead. Stated another way, the consequence is lost work time that amounts to a societal deficit that can be measured in billions of dollars and compared against the marginal cost of treatment. Improving measures of donor compatibility, shortening the time end-stage lung failure patients wait for a transplant and streamlining the donor referral process will only go so far towards reducing the risk of lung rejection; they will not eliminate it.

The impact of BOS is very different from other end-of-life diseases. The trends towards increasing numbers of transplants among a relatively young population suggest that burden of BOS will only continue to rise in the years ahead. According to the US Organ Procurement and Transplantation Network at HRSA there have been

604,434 lung transplants performed in the United States, with 68 active lung transplant programs currently underway (as of 25 July 2014). The 1653 patients who are actively awaiting lungs as of 1st August are in addition to the more than 10 000 people living with a transplanted lung (HRSA). According to the same registry, there were double the number of lung transplants performed in 2013 as compared with just over a decade earlier (1923 vs. 959 in 2000; adjusted mean age 45.4 years).[35]

## CONCLUSIONS

BOS will continue to present a substantial economic burden to society that extends far beyond its direct healthcare cost, due in large part to the foreshortened departure of thousands from the paid workforce. This rising burden of illness surrounding BOS is likely to receive greater attention given the movement towards precision medicine and wider societal pressure to contain costs. As with any other disease that threatens the lives and livelihoods of hitherto active members of society and their families, BOS should be viewed in its totality to include both the wider human capital perspective and a multi-year time horizon.

## ACKNOWLEDGMENTS

DGC is a recipient of a CJ Martin Fellowship from the National Health and Medical Research Council of Australia (# 1053790). CAJ is principal of ForMyOdds LLC a corporation that received sponsorship from Therakos Inc to perform inter alia this analysis. RV is supported by the Research Foundation Flanders (FWO) (KAN2014 1.5.139.14) and Klinisch Onderzoeksfonds (KOF) KU Leuven.
